# Incidence and risk factors of abnormal liver function and anti-tuberculosis drug-induced liver injury: a multicenter retrospective study involving 482 chinese pediatric tuberculosis patients

**DOI:** 10.1080/07853890.2025.2546701

**Published:** 2025-11-11

**Authors:** Yiqing Zhou, Min Wang, Hongmei Xu, Lisu Huang, Ping Liu, Juan Ma, Haiyan Li, Xin Yu, Qingshan Cai, Man Tian, Qing Fang, Xianhui Zeng, Guangxu Yang, Tao Yang, Fei Wang, Bin Chen

**Affiliations:** ^a^ School of Public Health, Hangzhou Medical College, Hangzhou, Zhejiang, China; ^b^ Department of Tuberculosis Control and Prevention, Quzhou Center for Disease Control and Prevention, Quzhou, Zhejiang, China; ^c^ Division of Infectious Diseases, Chongqing Medical University Affiliated Children’s Hospital, Chongqing, China; ^d^ Department of Infectious Diseases, The Children’s Hospital, Zhejiang University School of Medicine, National Clinical Research Center for Child Health, Hangzhou, Zhejiang, China; ^e^ Department of Tuberculosis, Shanghai Public Health Clinical Center, Fudan University, Shanghai, China; ^f^ Department of Infectious Diseases, Qinghai Province Women and Children’s Hospital, Xining, Qinghai, China; ^g^ Department of Pediatric Pulmonology, The Second Affiliated Hospital and Yuying Children’s Hospital of Wenzhou Medical University, Wenzhou, Zhejiang, China; ^h^ Department of Tuberculosis, Suzhou No. 5 People’s Hospital, Suzhou, Jiangsu, China; ^i^ Department of Tuberculosis, Hangzhou Red Cross Hospital, Hangzhou, Zhejiang, China; ^j^ Department of Respiratory Medicine, Children’s Hospital of Nanjing Medical University, Nanjing, Jiangsu, China; ^k^ Department of Pulmonary Medicine, Ningbo No. 2 Hospital, Ningbo, Zhejiang, China; ^l^ Department of Infectious Diseases, Hainan Women and Children’s Medical Center, Haikou, Hainan, China; ^m^ Tuberculosis Prevention Institute, Changchun Infectious Disease Hospital, Changchun, Jilin, China; ^n^ Scientific research center, Zhejiang SUKEAN Pharmaceutical Co. LTD, Hangzhou, Zhejiang, China; ^o^ Department of Tuberculosis Control and Prevention, Zhejiang Provincial Center for Disease Control and Prevention, Hangzhou, Zhejiang, China

**Keywords:** Tuberculosis, pediatric, liver injury, risk factor, anti-tuberculosis drug

## Abstract

**Objective:**

This study aimed to investigate the incidence of abnormal liver function, which can lead to serious consequences, as well as anti-tuberculosis drug-induced liver injury (ATDILI) and their associated risk factors.

**Methods:**

A national multicenter retrospective study was conducted to collected pediatric TB treatment data from 11 specialized and general hospitals. The multivariate logistic regression analysis was performed to assess the influencing factors of abnormal liver function and ATDILI. The Kaplan-Meier survival analysis assessed temporal risk trends for these outcomes.

**Results:**

Among the 482 patients included in the study, 18 patients (3.73%) experienced ATDILI, and 51 (10.58%) experienced abnormal liver function. The time of occurrence of abnormal liver function and ATDILI was 20.0 (IQR: 6.0, 34.0) days and 28.0 (IQR: 8.0, 87.0) days, respectively. The multivariate analysis results showed that, patients who used second line drugs in the intensive phase were more likely to experience ATDILI (OR = 4.16, 95% CI: 1.34 ∼ 12.84, *p* = 0.013). Patients with severe TB and who used the second line drugs were more likely to develop abnormal liver function (OR = 2.31, 95% CI: 1.08 ∼ 4.97, *p* = 0.031 for severity; OR = 3.06, 95% CI: 1.46 ∼ 6.43, *p* = 0.003 for treatment).

**Conclusion:**

The incidence of abnormal liver function and ATDILI differed. By considering laboratory indicators and clinical practice, early identification and detection of abnormal liver function in children during anti-TB treatment, along with timely intervention, can effectively prevent and control the harm caused by abnormal liver function and prevent it progression to ATDILI.

## 1. Introduction

Tuberculosis (TB) remains a major challenge to global public health. According to a 2024 global TB report, there were approximately 10.8 million TB cases worldwide in 2023, with an incidence of 134/100,000. The number of deaths owing to TB was 1.25 million. There were approximately 1.3 million cases of TB in children aged 0–14 years, accounting for 12% of the global burden of TB, and 190,000 deaths, accounting for 15% of TB deaths. The estimated number of new patients with TB in China is 741,000, of whom children aged 0–14 years accounted for approximately 5% [1].

TB is preventable and treatable in all age groups, including children [2]. For drug-susceptible TB, the WHO-recommended intensive treatment regimen includes isoniazid (H), rifampicin (R), pyrazinamide (Z), and ethambutol (E) for 2 months in the intensive phase, and H and R for 4 months in the continuous phase. In addition, in 2022, the WHO recommended a 4-month regimen (2HRZ(E)/2HR) for children and adolescents aged 3 months to 16 years with non-severe TB [3]. Although the recommended drug regimens are effective, safe, and well-tolerated in children, drug therapy has the potential for adverse reactions (ADRs) and adversely affects treatment adherence and prognosis in patients with TB [4].

Abnormal liver function always occurs during anti-TB treatment. When abnormal liver function persists, it may progress to anti-TB drug-induced liver injury (ATDILI). ATDILI is one of the most common and serious ADRs that occur during TB treatment [5,6]. In adults, host factors such as age; viral hepatitis; comorbidities with other acute and chronic liver diseases; HIV infection; malnutrition; severity of illness; drug factors such as H, R, Z, protionamide, and para-amino salicylic acid; and, other factors such as excessive alcohol consumption are associated with ATDILI development [7–16]. Compared to those in adults, there are fewer studies on the incidence and related influencing factors of ATDILI in children. Although the incidence of ATDILI in children is lower than that in adults, ATDILI can occur in any age group or in children receiving any dose of anti-TB drugs [17]. In many cases, the symptoms and signs of ATDILI are often ignored, and data on Chinese children with ATDILI are limited. The incidence of ATDILI in children, both domestically and internationally, ranges from 1.7 to 26.8% [2,5,17–23]. In contrast, the differences in ATDILI incidence reported in the literature may be related to factors such as ethnicity, socioeconomic status, geographic location, the researcher’s definition of ATDILI, study design, and sample size.

Abnormal liver function and ATDILI is usually observed during the initial phase of anti-TB treatment. It not only poses a serious diagnostic and management challenge for pediatricians but also makes resumption of treatment difficult if the specific drugs are temporarily discontinued and substituted with safer drugs [17]. Therefore, this study aimed to investigate the incidence of abnormal liver function and ATDILI, and their associated factors during anti-TB treatment in pediatric patients in a multicenter study.

## 2. Patients and methods

### 2.1. Study design

The study was conducted in 11 regionally representative children’s specialist or general hospitals that offered TB treatment in seven provinces of China. This was a multicenter retrospective study that collected data on TB cases among children in these hospitals. Hospital information systems (HIS), laboratory information systems (LIS), Picture Archiving and Communication Systems (PACS), and other system queries were used to collect basic patient information, medication information, and information on ATDILI incidence.

According to socioeconomic development, future development strategy, and geographical location, we divided the provinces where several hospitals were located into three regions: east, west, and northeast. The eastern region included Shanghai, Jiangsu, Zhejiang, and Hainan; west, Chongqing, and Qinghai; and, Northeast, Jilin.

### 2.2. Patients

All pediatric patients with TB were treated with H, R, and Z in at least three drugs during the intensive phase, with doses based on the pretreatment body weight. Subsequent treatments with H and R in at least two drugs were used in the continuation phase. And all included patients had microbiologically or clinically confirmed drug-susceptible TB. Patients who were ≤14 years old and weighed <40 kg at the time of enrolment were included. In addition, we extracted complete core information of the patients at baseline and clinical follow-up. We excluded neonates who were aged ≤1 month and weighed ≤4 kg at the time of inclusion.

### 2.3. Data collection

Data collection information included sex; age; site; severity of TB; doses of H, R, and Z during the intensive period; treatment regimen during the intensive period; and, specifics of the occurrence of abnormal liver function and ATDILI.

### 2.4. Definition

#### 2.4.1. TB

Patients diagnosed with active TB based on the classification criteria for TB (WS196-2017) and TB diagnosis (WS288-2017) were included.

#### 2.4.2. Severe TB

Non-severe TB was defined as TB of the peripheral lymph nodes; TB of the intrathoracic lymph nodes without airway obstruction; and tuberculous pleural effusion or oligomicrobial noncavitary disease without complications, confined to the lobes of the lungs, without cornified changes [24]. Patients were considered to have severe TB if they did not present with any of these conditions.

#### 2.4.3. Malnutrition

When height and weight data of children were available, Z-scores were calculated for their BMI-for-age, and they were defined as malnourished if their Z-scores were ≥2 standard deviations (SD) below the mean reference value. If only weight was reported, the Z-score for weight-for-age was calculated; if it was ≥2 SD below the mean reference value, it was considered as malnutrition.

#### 2.4.4. Abnormal liver function, ATDILI, and severity of abnormal liver function and ATDILI

Liver enzyme levels were monitored regularly by tests conducted in local laboratories after treatment or according to the judgment of clinicians. All elevations of alanine transaminase (ALT), aspartate transaminase (AST), alkaline phosphatase (ALP), and total bilirubin (TBil) levels were based on the upper limit of the normal (ULN) threshold standardized by a local reference laboratory. Based on the laboratory test results, the situation where any of the indicators of ALT, AST, ALP, and TBil exceeds the upper limit of the ULN was defined as abnormal liver function [25]. Based on expert discussions and clinical doctors’ judgments, the severity levels were categorized into three tiers according to the impact of the severity of abnormal liver function on treatment. Regarding the severity stratification (mild/moderate/severe) based on treatment impact, we implemented specific measures to minimize subjective variability among clinicians. Prior to data analysis, clear severity stratification standards are formulated through the collaboration of an expert panel. For each patient with laboratory-confirmed liver function abnormalities (ALT/AST/ALP/TBil > ULN), two panel members (pediatric infectious disease and TB doctors) first independently evaluated the case, considering both the laboratory data and clinical context (e.g. symptoms, treatment progress) to determine the severity tier. If the two reviewers reached different conclusions, a third panel member conducted a blinded re-evaluation of the case, resolving the discrepancy based on the predefined criteri. The specific classification is as follows: Level 1 (mild), in which the original anti-TB regimen was maintained; Level 2 (moderate), which required adjustments to the anti-TB treatment; and level 3 (severe), leading to the termination of anti-TB therapy.

Considering that there is no expert consensus or guideline for the diagnosis and treatment of ATDILI in children, we evaluated the disease according to the guidelines for the diagnosis and management of drug-induced liver injury caused by anti-TB drugs (2024) [26]. The severity levels were defined as follows: Level 1 (mild) characterized by serum ALT ≥3 ULN and/or ALP ≥2 ULN, with TBil <2 ULN; Level 2 (moderate), serum ALT ≥5 ULN and/or ALP ≥2 ULN, with TBil ≥2 ULN, accompanied by possible hepatitis symptoms; Level 3 (severe), serum ALT ≥5 ULN and/or ALP ≥2 ULN, with TBil ≥2 ULN, presenting with hepatitis symptoms alongside any of the following: INR ≥1.5; ascites and/or hepatic encephalopathy; other organ failure due to drug-induced liver injury (DILI); and, Level 4 (fatal), death from DILI or the necessity of liver transplantation. The grading criteria were based on the International DILI Expert Working Guidelines, American Thoracic Society Guidelines, Asia-Pacific Liver Society Consensus Guidelines 2021, American Academy of Liver Diseases Practice Guidelines 2022, and Chinese Guidelines for the Diagnosis and Management of Drug-induced Liver Injury 2023 [27–30].

#### 2.4.5. Dosage classification

The high-dose group comprised cases in which at least one of the daily doses per kilogram or the daily maximum dose exceeded the upper limit, whereas the non-high-dose group comprised cases in which both the daily dose per kilogram and the daily maximum dose remained within the recommended limits. In particular, the dosing details of H, R, and Z were as follows: H had a daily dose of 10 (7–15) mg/kg, with a maximum daily dose of 300 mg for individuals weighing <50 kg and a conventional administration frequency of once daily; R was administered at 15 (10–20) mg/kg daily, with a maximum dose of 600 mg for those weighing <50 kg, with a once-daily regimen; Z had a daily dose of 35 (30–40) mg/kg, with no specified daily maximum dose for individuals weighing <50 kg, and a standard administration frequency of once daily.

### 2.5. Statistical analysis

All analyses were conducted using R version 4.4.1. Categorical variables were expressed as frequencies (percentages) when descriptive statistics was used. We used univariate and multivariate logistic regression to explore the influencing factors related to the occurrence of abnormal liver function and ATDILI. Differences were considered statistically significant when the P-value was <0.05. To assess the changing trend of the risk of abnormal liver function and ATDILI in the study population over time, we conducted a survival analysis. Specifically, we adopt the Non-parametric Kaplan-Meier method to estimate the Cumulative Hazard Function.

### 2.6. Ethics approval and consent to participate

The study was performed according to the guidelines of the Declaration of Helsinki. The studies involving human participants were reviewed and approved by The Medical Ethical Committee of Zhejiang Provincial Center for Disease Control and Prevention (Approval number: 2023-018-01). As the study only involved the collection of medical record data and did not include personal identifying information, it posed no risks or adverse effects on the rights and health of the subjects. Therefore, The Medical Ethical Committee of Zhejiang Provincial Center for Disease Control and Prevention granted an informed consent waiver.

## 3. Results

### 3.1. Patients

Totally 482 pediatric patients with TB were included (Table 1). Of these, 53.73% (*n* = 259) were men. In terms of age stratification, 39.21% (*n* = 189) of the children were aged 0–4 years; 35.48% (*n* = 171), 5–9 years; and, 25.31% (*n* = 122), 10–14 years. Among pediatric patients with TB, 15.15% (*n* = 73) were malnourished. Most patients had PTB (94.19%, *n* = 454). Moreover, most pediatric patients (63.69%, *n* = 307) were diagnosed with severe TB. The treatment options for children with TB mainly include first-line-only regimen (HRZE/HRZ) or first-line regimen augmented with second-line drugs. Most patients (87.76%, *n* = 423) chose first-line drug treatment in the intensive phase, that is HRZ or HRZE. A few patients used first-line regimen augmented with second-line drugs such as linezolid and levofloxacin. In addition, we excluded cases in which first-line anti-TB drugs were replaced with second-line drugs because of ATDILI. Some patients used high doses of H (4.77%, *n* = 23), R (2.07%, *n* = 10), and Z (3.73%, *n* = 18) along with their medications. During the intensive phase of treatment, H exceeded the daily dose per kg or daily maximum dose limit in 23 cases, and of the 16 cases in which the dose exceeded 15 mg/kg, the median dose was 16.67 (IQR: 15.75–17.73) mg/kg, and 7 patients had a daily maximum dose of >300 mg, with doses between 350 and 450 mg. Regarding R, 10 patients exceeded the upper limit of daily dose per kg when using R, and the median dose was 21.96 (IQR: 21.43–27.06) mg/kg. Regarding Z, 18 patients exceeded the upper limit of daily dose per kg, and the median dose was 47.48 (IQR: 42.86–50.00) mg/kg. Some children with TB were treated with a combination of drugs (18.05%, *n* = 87), including meropenem, azithromycin, and cephalosporins. Combination medications were administered to patients with documented or suspected bacterial co-infections (e.g. pneumonia, sepsis) based on clinical symptoms, laboratory markers (e.g. elevated CRP, leukocytosis), or imaging findings.

**Table 1.** Baseline characteristics including clinical features of patients by different definitions.

Variables	Total (*n* = 482)	ATDILI		Abnormal liver function	
		No (*n* = 464)	Yes (*n* = 18)	No (*n* = 431)	Yes (*n* = 51)
Sex					
Male	259 (53.73)	251 (96.91)	8 (3.09)	232 (89.58)	27 (10.42)
Female	223 (46.27)	213 (95.52)	10 (4.48)	199 (89.24)	24 (10.76)
Age					
0–4 years old	189 (39.21)	179 (94.71)	10 (5.29)	164 (86.77)	25 (13.23)
5–9 years old	171 (35.48)	166 (97.08)	5 (2.92)	154 (90.06)	17 (9.94)
10–14 years old	122 (25.31)	119 (97.54)	3 (2.46)	113 (92.62)	9 (7.38)
Malnutrition					
No	409 (84.85)	393 (96.09)	16 (3.91)	364 (89.00)	45 (11.00)
Yes	73 (15.15)	71 (97.26)	2 (2.74)	67 (91.78)	6 (8.22)
Site					
PTB	454 (94.19)	437 (96.26)	17 (3.74)	408 (89.87)	46 (10.13)
EPTB	28 (5.81)	27 (96.43)	1 (3.57)	23 (82.14)	5 (17.86)
Severity of TB					
Non-severe	175 (36.31)	171 (97.71)	4 (2.29)	165 (94.29)	10 (5.71)
Severe	307 (63.69)	293 (95.44)	14 (4.56)	266 (86.64)	41 (13.36)
Isoniazid					
Non-high dose	459 (95.23)	442 (96.30)	17 (3.70)	409 (89.11)	50 (10.89)
High dose	23 (4.77)	22 (95.65)	1 (4.35)	22 (95.65)	1 (4.35)
Rifampicin					
Non-high dose	472 (97.93)	455 (96.40)	17 (3.60)	423 (89.62)	49 (10.38)
High dose	10 (2.07)	9 (90.00)	1 (10.00)	8 (80.00)	2 (20.00)
Pyrazinamide					
Non-high dose	464 (96.27)	446 (96.12)	18 (3.88)	415 (89.44)	49 (10.56)
High dose	18 (3.73)	18 (100.00)	0 (0.00)	16 (88.89)	2 (11.11)
Treatment					
First-line-only regimen (HRZE/HRZ)	423 (87.76)	411 (97.16)	12 (2.84)	386 (91.25)	37 (8.75)
Frst-line regimen augmented with second-line drugs	59 (12.24)	53 (89.83)	6 (10.17)	45 (76.27)	14 (23.73)
Combination medication					
No	395 (81.95)	380 (96.20)	15 (3.80)	354 (89.62)	41 (10.38)
Yes	87 (18.05)	84 (96.55)	3 (3.45)	77 (88.51)	10 (11.49)
Region					
Eastern	320 (66.39)	307 (95.94)	13 (4.06)	290 (90.62)	30 (9.38)
Western	157 (32.57)	152 (96.82)	5 (3.18)	136 (86.62)	21 (13.38)
Northeast	5 (1.04)	5 (100.00)	0 (0.00)	5 (100.00)	0 (0.00)

### 3.2. Incidence and clinical characteristics of patients with abnormal liver function and ATDILI

Among the 482 patients included in our study, 51 (10.58%) patients experienced abnormal liver function at a median of 28.0 (IQR: 8.0–87.0) days, with a duration of 28.0 (IQR: 13.0–56.0) days from abnormal liver function onset to resolution (Table 2). Furthermore, 38 patients developed mild abnormal liver function, and 11 and 2 developed moderate and severe abnormal liver function, respectively. Most patients (66.67%, *n* = 34) developed abnormal liver function within 56 days. Among the 51 patients with abnormal liver function, there were 31 cases where AST exceeded ULN, 34 cases where ALT exceeded ULN, 8 cases where ALP exceeded ULN, and 8 cases where TBil exceeded ULN.

**Table 2.** Clinical characteristics of 18 ATDILI patients by different definitions.

Variables	ATDILI (*n* = 18)	Abnormal liver function (*n* = 51)
Days between initial TB treatment and ATDILI/abnormal liver function	20.0 (6.0, 34.0)	28.0 (8.0, 87.0)
Duration of ATDILI//abnormal liver function recognition	28.0 (15.0, 55.0)	28.0 (13.0, 56.0)
Days between initial TB treatment and moderate to severe ATDILI/abnormal liver function	28.0 (18.0, 32.0)	28.0 (8.0, 36.0)
Duration of moderate to severe ATDILI//abnormal liver function recognition	28.0 (14.0, 28.0)	28.0 (14.0, 56.0)
Severity of ATDILI/abnormal liver function		
Mild ATDILI/abnormal liver function	11 (61.11)	38 (74.51)
Moderate ATDILI/abnormal liver function	7 (38.89)	11 (21.57)
Severe ATDILI/abnormal liver function	0 (0.00)	2 (3.92)
The number of other adverse reaction		
No	12 (66.67)	32 (62.75)
One	4 (22.22)	16 (31.37)
Two	1 (5.56)	2 (3.92)
Three	1 (5.56)	1 (1.96)

There were 18 (3.73%) patients developed ATDILI at a median of 20.0 (6.0, 34.0) days after the initiation of anti-TB treatment, with a duration of 28.0 (IQR: 15.0–55.0) days from ATDILI onset to resolution (Table 2). 11 patients presented with mild ATDILI, which did not affect treatment. 7 patients presented with moderate ATDILI, and none experienced severe ATDILI or death. In addition, 6 patients experienced other ADRs, including hematologic impairment, nausea and vomiting, gastrointestinal impairment, psychiatric symptoms, and dermatologic disorders. Most patients (77.78%, *n* = 14) developed ATDILI within 56 days of starting treatment, whereas 13 patients (72.22%) developed ATDILI within 28 days. Among the 18 patients with ATDILI, 17 (94.44%) had acute ATDILI, and only 1 (5.56%) had chronic ATDILI in the 12th month of treatment. Among the 18 patients with ATDILI, 12 (66.67%) had hepatocellular ATDILI, mainly with elevated ALT levels; 4 (22.22%), cholestatic ATDILI, mainly with elevated ALP levels; and, 2 (11.11%), mixed ATDILI, and ALT and ALP levels exceeded the specified indicators. None of the patients in our study had liver failure or life-threatening ATDILI.

**Figure 1.**
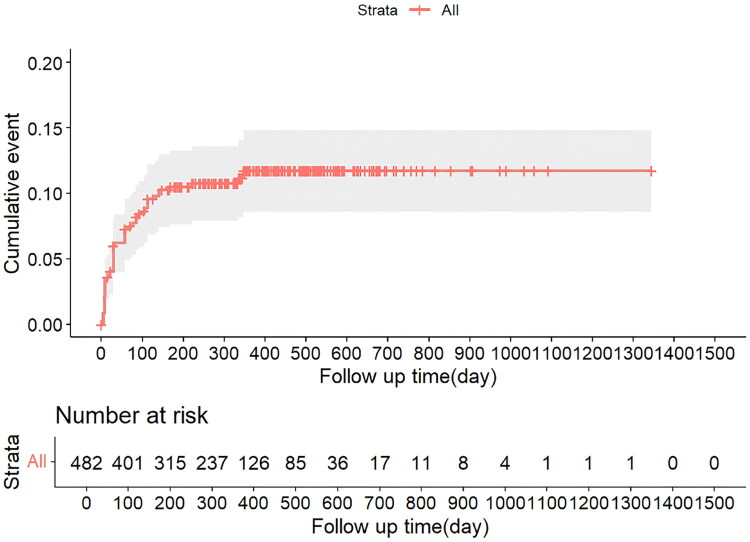
Cumulative hazard plot of abnormal liver function among children TB patients.

**Figure 2.**
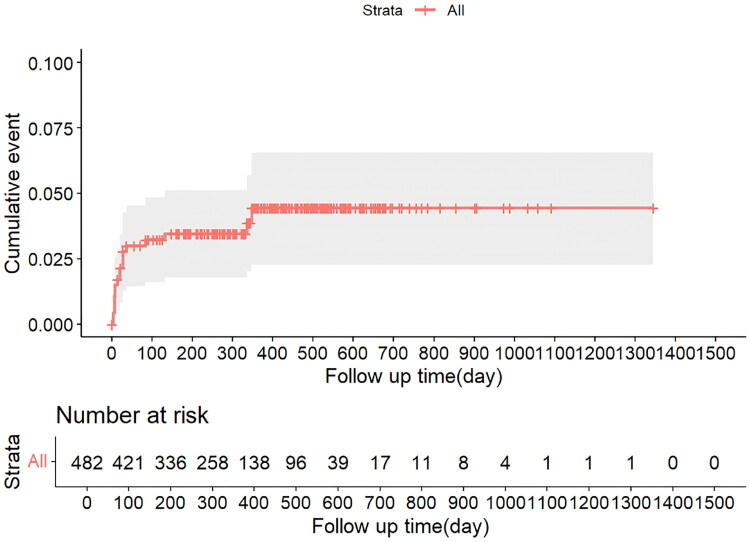
Cumulative hazard plot of ATDILI among children TB patients.

Figures 1 and 2 showed a sharp decline in the number of children with TB at risk of abnormal liver function and ATDILI over the follow-up period, especially in the first 100 days when the at-risk population decreased most rapidly, suggesting that abnormal liver function and ATDILI mainly occurs in the early stage of treatment.

### 3.3. Risk factors for abnormal liver function and ATDILI

The results of the univariate and multivariate logistic regression analyses of the risk factors associated with abnormal liver function in this study were presented in Table 3. Patients with severe TB were more likely to have abnormal liver function compared with patients with non-severe TB (OR = 2.31, 95% CI: 1.08 ∼ 4.97, *p* = 0.031). Pediatric patients with TB treated with first-line regimen augmented with second-line drugs during the intensive phase were 3.06 times more likely to develop abnormal liver function than those treated with first-line-only regimen (HRZE/HRZ) (95% CI: 1.46 ∼ 6.43, *p* = 0.003).

**Table 3.** Risk factors associated with abnormal liver function by univariate and multivariate logistic regression analysis based on expert judgment.

Variables	Univariate		Multivariate	
	OR (95% CI)	*P*	OR (95% CI)	*P*
Sex				
Male	Reference		Reference	
Female	1.04 (0.58 ∼ 1.85)	0.904	1.16 (0.63 ∼ 2.14)	0.639
Age				
0–4 years old	Reference		Reference	
5–9 years old	0.72 (0.38 ∼ 1.39)	0.334	0.71 (0.35 ∼ 1.42)	0.329
10–14 years old	0.52 (0.24 ∼ 1.16)	0.111	0.51 (0.22 ∼ 1.19)	0.120
Malnutrition				
No	Reference		Reference	
Yes	0.72 (0.30 ∼ 1.77)	0.478	0.78 (0.30 ∼ 2.02)	0.614
Site				
PTB	Reference		Reference	
EPTB	1.93 (0.70 ∼ 5.32)	0.204	1.57 (0.54 ∼ 4.55)	0.408
Severity of TB				
Non-severe	Reference		Reference	
Severe	2.54 (1.24 ∼ 5.21)	**0.011**	2.31 (1.08 ∼ 4.97)	**0.031**
Isoniazid				
Non-high dose	Reference		Reference	
High dose	0.37 (0.05 ∼ 2.82)	0.338	0.15 (0.02 ∼ 1.53)	0.110
Rifampicin				
Non-high dose	Reference		Reference	
High dose	2.16 (0.45 ∼ 10.45)	0.339	3.57 (0.57 ∼ 22.57)	0.176
Pyrazinamide				
Non-high dose	Reference		Reference	
High dose	1.06 (0.24 ∼ 4.74)	0.941	1.14 (0.23 ∼ 5.64)	0.875
Treatment				
First-line-only regimen (HRZE/HRZ)	Reference		Reference	
Frst-line regimen augmented with second-line drugs	3.25 (1.63 ∼ 6.46)	**<.001**	3.06 (1.46 ∼ 6.43)	**0.003**
Combination medication				
No	Reference		Reference	
Yes	1.12 (0.54 ∼ 2.34)	0.760	0.71 (0.31 ∼ 1.63)	0.423
Region				
Eastern	Reference		Reference	
Western	1.49 (0.82 ∼ 2.70)	0.186	1.21 (0.62 ∼ 2.33)	0.577
Northeast	0.00 (0.00 ∼ Inf)	0.984	0.00 (0.00 ∼ Inf)	0.989

The results of the univariate and multivariate logistic regression analyses of the risk factors associated with ATDILI in this study were presented in Table 4. The variable of significance in the univariate results was the treatment regimen administered during the intensive phase. The subsequent multivariate logistic regression analysis showed that pediatric patients with TB treated with first-line regimen augmented with second-line drugs during the intensive phase were 4.16 times more likely to develop ATDILI than those treated with first-line-only regimen (HRZE/HRZ) (95% CI: 1.34 ∼ 12.84, *p* = 0.013).

**Table 4.** Risk factors associated with ATDILI by univariate and multivariate logistic regression analysis.

Variables	Univariate		Multivariate	
	OR (95% CI)	*P*	OR (95% CI)	*P*
Sex				
Male	Reference		Reference	
Female	1.47 (0.57 ∼ 3.80)	0.423	1.73 (0.64 ∼ 4.68)	0.279
Age				
0–4 years old	Reference		Reference	
5–9 years old	0.54 (0.18 ∼ 1.61)	0.268	0.59 (0.19 ∼ 1.86)	0.368
10–14 years old	0.45 (0.12 ∼ 1.67)	0.234	0.42 (0.11 ∼ 1.67)	0.220
Malnutrition				
No	Reference		Reference	
Yes	0.69 (0.16 ∼ 3.07)	0.628	0.68 (0.14 ∼ 3.29)	0.636
Site				
PTB	Reference		Reference	
EPTB	0.95 (0.12 ∼ 7.42)	0.963	0.61 (0.07 ∼ 5.24)	0.656
Severity of TB				
Non-severe	Reference		Reference	
Severe	2.04 (0.66 ∼ 6.30)	0.214	2.10 (0.63 ∼ 6.98)	0.224
Isoniazid				
Non-high dose	Reference		Reference	
High dose	1.18 (0.15 ∼ 9.29)	0.874	0.78 (0.07 ∼ 8.91)	0.845
Rifampicin				
Non-high dose	Reference		Reference	
High dose	2.97 (0.36 ∼ 24.82)	0.314	6.11 (0.49 ∼ 75.59)	0.158
Pyrazinamide				
Non-high dose	Reference		Reference	
High dose	0.00 (0.00 ∼ Inf)	0.988	0.00 (0.00 ∼ Inf)	0.992
Treatment				
First-line-only regimen (HRZE/HRZ)	Reference		Reference	
Frst-line regimen augmented with second-line drugs	3.88 (1.40 ∼ 10.76)	**0.009**	4.16 (1.34 ∼ 12.84)	**0.013**
Combination medication				
No	Reference		Reference	
Yes	0.90 (0.26 ∼ 3.20)	0.876	0.57 (0.14 ∼ 2.38)	0.440
Region				
Eastern	Reference		Reference	
Western	0.78 (0.27 ∼ 2.22)	0.637	0.55 (0.17 ∼ 1.74)	0.305
Northeast	0.00 (0.00 ∼ Inf)	0.990	0.00 (0.00 ∼ Inf)	0.996

## 4. Discussion

During the process of anti-TB treatment, abnormal liver function was also very common, usually manifested as elevated ALT and AST, among which ALT was the most sensitive indicator reflecting liver cell damage [31]. In our study, approximately 10% of the patients experienced abnormal liver function. Among these patients with abnormal liver function, one quarter of them adjusted their treatment plans or stopped treatment due to the occurrence of abnormal liver function. Among these patients, a small number did not reach the degree of ATDILI, but the clinicians still adjusted the treatment for children with TB based on the specific circumstances. This might be because, in the clinical practice process, most clinicians still comprehensively consider symptoms, treatment effects and imaging manifestations, etc., and do not mechanically consider that clinical measures can only be taken when ATDILI is achieved. When liver function indicators exceed the normal upper limit, taking corresponding measures is conducive to ensuring the smooth progress of anti-TB treatment. In addition, as the disease progresses, abnormal liver function in children with TB may develop into ATDILI [32]. Therefore, it is also very necessary to pay attention to the influencing factors that affect abnormal liver function. ATDILI in children mainly arises from cytotoxic injury triggered by drugs or their metabolites during anti-TB treatment in children or due to hepatic metabolic reactions to drugs and their metabolites. In our study, 3.73% of patients were categorized as having ATDILI, which was low compared with the incidence reported previously [2,5,17–23]. Additionally, the incidence was also lower compared to that in adults [33–36]. In 18 cases of ATDILI, most patients presented within 56 days of treatment, which was comparable to other studies [2,19,37]. In addition, patients with ATDILI in this study mainly had hepatocellular ATDILI, corroborating previous studies [38]. Furthermore, 72% patients developed ATDILI within 28 days of treatment, suggesting that clinicians should prescribe liver function tests to patients as early as possible during the treatment of pediatric TB, closely monitor liver function during medication administration for 56 days prior to treatment, and provide parents with detailed information about the possible adverse effects of anti-TB drugs and their clinical manifestations. This may help minimize the incidence of ATDILI by reducing parents’ and patients’ resistance to medication and excessive worry and anxiety about possible adverse effects and prompting them to undergo routine follow-up on time.

In our study, most pediatric patients had severe TB, likely due to the relatively insidious onset of TB in children, rapid onset of the disease, and tendency to develop blood-borne disseminated TB [39]. In addition, the TB diagnosis and treatment hospitals included in our study were provincial and municipal regional diagnosis and treatment centers that accepted admissions for more difficult and critical cases than those accepted by primary hospitals. Patients with severe TB usually have a long illness duration, malnutrition, and impaired immune function [14]. Our results did not show that pediatric patients with severe TB were more likely to develop ATDILI. Nevertheless, clinicians should closely monitor ADRs to adjust treatment strategies in a timely manner in pediatric patients with severe TB. Most importantly, we need to strengthen the education of the public, particularly parents, to raise awareness of childhood TB, promote early medical consultation and standardized treatment, and reduce the transmission of TB and the incidence of severe cases. However, based on abnormal liver function results judged by the laboratory indicators and actual clinical situation, patients with severe TB were more likely to develop abnormal liver function. This may be due to the clinical complexity or other comorbidities in patients with severe TB. Even if the laboratory parameters are mildly elevated (not reaching two or three times the ULN), if it is accompanied by jaundice, abnormal coagulation function, or symptoms of nausea and fatigue, clinicians will judge it as an abnormal liver function case after comprehensive consideration and perform timely monitoring and intervention. Therefore, in clinical practice, it is necessary to balance guideline standards and drug safety in children and perform early interventions for high-risk patients, such as children with severe TB, to reduce the incidence of abnormal liver function to further lower the incidence of ATDILI.

Patients treated with second-line drugs were more likely to experience ADRs, including ATDILI, which was consistent with our findings [22,40]. Pediatric patients with TB who were treated with first-line drugs in combination with second-line drugs during the intensive phase were more likely to develop ATDILI. The second-line drugs used in the present study were linezolid and levofloxacin. Linezolid is metabolized by the liver, and if a patient develops hepatic insufficiency during anti-TB treatment, resulting in the inability of the liver to properly metabolize the drug, the drug may accumulate in the body, increasing blood levels and the risk of adverse reactions [41]. ATDILI might also occur if the patient’s medication is prolonged or if the dose of the medication is increased owing to a complex medical condition or for other reasons. The efficacy of levofloxacin, a fluoroquinolone antimicrobial drug, in combination with other anti-TB drugs for the treatment of TB has been clinically proven. In patients with drug-susceptible TB, levofloxacin may be considered when first-line drugs are not effective [42]. Levofloxacin has a long elimination half-life, improved tissue penetration, and safe efficacy. Although a few studies have reported levofloxacin-induced liver injury, complex conditions leading to prolonged levofloxacin use can lead to abnormal liver function. Therefore, a detailed understanding of the basic data of children in the clinic is required along with improved monitoring of liver and kidney function and blood routine during the course of anti-TB treatment for children with risk factors for ATDILI to adjust the treatment program in time, thus improving the prognosis. In addition, our results showed that the use of high doses of H, R, or Z during the intensification period did not cause ATDILI. This may be because the doses of the drugs were not yet very high or possibly did not reach the level of injury during clinical use.

Previous findings have shown that gender was an influential factor in patients presenting with ATDILI [43,44]. In children with incomplete development of secondary sexual characteristics, differences in male and female physiology and functioning between the sexes were not obvious. However, when they reach adulthood, sexual development is complete, and there were large differences in their physical characteristics. In addition, age has been shown to be a high-risk factor for ATDILI [45]. This may be related to the declining physical functions of older people, whose physiological functions decline, drug metabolism slows down, and liver regenerative functions were also affected [46,47]. However, Gafar et al. found that age was not a factor influencing the development of ATDILI in pediatric patients with TB, which was consistent with the results of our study [19]. This may be because the hepatic metabolic function in children was usually relatively stable at this stage and hads not reached the full maturity level of that of adults [48]. This meant that the rate of metabolism of the drug was likely to be relatively consistent and not significantly different depending on age. In addition, common risk factors associated with the development of ATDILI in adults, such as comorbid diabetes, HIV infection, smoking, and alcohol consumption, were not included in this study because they are extremely rare in pediatric patients with TB.

Our study had some limitations. First, our study had a small sample size, which may have weakened its statistical validity. Second, this was a retrospective study, and confounding factors could not be completely excluded. However, our study had some strengths. First, we conducted a multicenter study covering multiple regions of the country, which, to some extent, reflects the current status of childhood TB treatment in China. Second, this was one of the first studies in China to utilize data from multiple hospitals spanning a 6-year period, which has some research significance.

## 5. Conclusion

In this study, the incidence of ATDILI was relatively low, but the incidence of abnormal liver function was relatively high. Most patients develop abnormal liver function and ATDILI within the first two months of anti-TB treatment, and this calls for targeted intensification of monitoring and management—particularly for severe cases and those on second-line drugs. This emphasizes the importance of intensive monitoring of biomarkers related to abnormal liver function and ATDILI and integrated clinical assessment in the initial treatment stage for early identification and timely intervention.

## Data Availability

The data that support the findings of this study are available from the corresponding author [Fei Wang] upon reasonable request.
